# Primary Immunodeficiency Diseases at Reference and High-Specialty Hospitals in the State of Guanajuato, Mexico

**DOI:** 10.1155/2013/187254

**Published:** 2013-09-01

**Authors:** Eduardo Guaní-Guerra, Ulises Noel García-Ramírez, Ana Isabel Jiménez-Romero, José Manuel Velázquez-Ávalos, Gabriela Gallardo-Martínez, Francisco-Javier Mendoza-Espinoza

**Affiliations:** ^1^Departamento de Inmunología, Hospital Regional de Alta Especialidad del Bajío, Boulevard Milenio 130 San Carlos La Roncha, 37660 León, GTO, Mexico; ^2^Centro Médico Nacional del Bajío, IMSS, 37320 León, GTO, Mexico; ^3^Departamento de Inmunoalergología Pediátrica, Hospital Ángeles-León, 37150 León, GTO, Mexico; ^4^Hospital Regional de León ISSSTE, 37520 León, GTO, Mexico

## Abstract

*Background*. In general, primary immunodeficiency diseases (PIDs) are underdiagnosed in most countries. The objective of this study was to describe the frequency and clinical spectrum of PID in the most important tertiary hospitals in our region. *Methods*. An observational, cross-sectional, with retrospective chart, review study was conducted. A total of 26 patients were included and grouped according to the updated classification of PIDs. *Results*. PIDs spectra were as follows: predominantly antibody deficiency diseases were the most common category (65.38%), followed by other well-defined immunodeficiency syndromes (11.55%), congenital defects of phagocyte number and/or function (7.69%), complement deficiencies (3.85%), combined T- and B-cell immunodeficiencies (3.85%), and defects in innate immunity (3.85%). The mean time elapsed from the onset of symptoms to the reference and diagnosis by a tertiary hospital was of 4.65 ± 6.95 years. *Conclusions*. Predominant antibody deficiency disease was the most common group of PIDs, agreeing with international reports. Awareness of underdiagnosis by physicians is crucial for a prompt diagnosis and treatment, which in turn should improve the quality of life among patients with PIDs.

## 1. Introduction

Primary immunodeficiencies (PIDs) are a group of diseases caused by inherited defects of the immune system, in which the common hallmark is the susceptibility to infection. Nowadays, more than 180 different PIDs have been described [1, 2].

However, research on PIDs has not been easy and still faces an array of difficulties. These are due in part to the fact that PIDs were discovered only half a century ago and are still a somewhat new area for common medicine. In some countries, such as Bulgaria, PIDs are even not acknowledged as disease entities and therefore cannot be diagnosed and treated accordingly [3]. Other challenges regarding PIDs include the broad range of diseases caused by a large variety of genetic defects, clinical variations in presentation, complexity of laboratory and genetic assessment, and costs of therapy [4].

While once thought to be exceedingly rare, symptomatic primary immunodeficiencies are now appreciated to range from 1 : 500 to 1 : 500,000 in the general population in the United States and Europe [5, 6]. A random digit dialing telephone survey in 2007 estimated that one in 1200 people within the United States are diagnosed with an immunodeficiency [7]. If one were to view immunodeficiency disorders as genetic in etiology, this statistic would rank PIDs as more common in the United States than some better-known genetic disorders, such as hemophilia (less than 15,000), cystic fibrosis (30,000), and Huntington disease (30,000) [8].

Unfortunately, in Mexico, we lack studies that show the prevalence, incidence, and burden cost of PIDs in our population. The objective of the present study is to collect and analyze data on patients with PID in the state of Guanajuato in Mexico, to facilitate diagnosis, treatment, research, and education of such patients, as the first step to improve their quality of life.

## 2. Materials and Methods

An observational, cross-sectional, with retrospective chart review study was conducted in four different reference centers or tertiary referral hospitals in the state of Guanajuato, Mexico. A total of 26 patients were included and grouped according to the updated classification of PIDs introduced by the Expert Committee of International Union of Immunological Societies (IUIS) on Primary Immunodeficiency [1]. The study subjects included in the present survey were patients from the immunology department of the different reference centers. At the moment of the study the total amount of patients in the four reference centers was estimated to be 344,672 people. The four high-specialty hospitals have a mean of 86,168 active patients; 147 hospital beds; 9,126 admissions per year; and 9,004 outpatients per year. The patients' parents were considered to be related if parental consanguinity was of the first or second degree. The study was approved by the institutional ethics committee and the institutional review board on clinical investigation. Quantitative variables were described using mean ± standard deviation (SD) and categorical data using absolute and relative frequencies. Statistical analysis was performed using the Sigmaplot v.11.0 software for Windows.

## 3. Results

As mentioned before, the sample included 26 patients, 11 females (42.3%) and 15 males (57.7%). The male-to-female ratio was 1.36 : 1. The mean age at the moment of the study was 12.35 ± 11.8 years (*n* = 23). At the moment of diagnosis, 25 patients (96.15%) were under the age of 16 years, and the mean age reported at that time was 8.2 ± 10.93 years. Among the 23 living patients, 20 (86.96%) were aged 15 years or younger and 3 (13.04%) patients were aged 16 years or older. The mean time which elapsed from the onset of symptoms to the reference and diagnosis into a tertiary hospital was of 4.65 ± 6.95 years. The overall mortality rate was 11.54% (*n* = 3). In 10 patients (43.48%) complications secondary to the PID were detected. Among these, bronchiectasis and chronic lung disease secondary to recurrent lung infections were the most common (*n* = 8/10, 80%). See Table 1 for general characteristics of studied patients.

PID spectra were as follows: predominantly antibody deficiency diseases (65.37%), well-defined immunodeficiency syndromes (11.55%), congenital defects of phagocyte number and/or function (7.69%), complement deficiencies (3.85%), combined immunodeficiencies (3.85%), and defects in innate immunity (3.85%). The well-defined syndromes are a group of diseases in which the occurrence of signs and symptoms point towards PID in patients with syndromic features. Examples of such diseases are the Wiskott-Aldrich syndrome (WAS), ataxia-telangiectasia, and DiGeorge anomaly [1]. Among the predominantly antibody deficiency diseases, the most frequent were the common variable immunodeficiency disorders (8/17, 47%), followed by X-linked agammaglobulinemia (3/17, 17.6%), selective IgA deficiency (2/17, 11.8%), isolated IgG subclass deficiency (2/17, 11.8%), and transient hypogammaglobulinemia of infancy with normal numbers of B cells (2/17, 11.8%); see Tables 2 and 3 for the PID spectra, clinical characteristics of studied patients, and etiological agents. The IgG levels in patients with common variable immunodeficiency disorders and X-linked agammaglobulinemia (XLA) at the moment of diagnosis were 151 ± 87.18 mg/dL and 413.71 ± 205.59 mg/dL, respectively. The mean serum levels after intravenous immunoglobulin replacement therapy (IRT) or trough serum levels were 1115.5 ± 218.5 mg/dL for XLA and 1434.13 ± 527.86 mg/dL for common variable immunodeficiency disorders (CVID) patients. The interval of intravenous immunoglobulin (IVIG) administration among the different hospitals and reference centers ranged from 3 to 4 weeks, and dosing of IVIG varied from 500 mg/dL to 700 mg/dL. All patients with CVID (*n* = 8) and XLA (*n* = 3) were under IVIG replacement therapy. First-degree parental consanguinity was observed only in one case. No family history of primary immunodeficiency disease was reported.

Regarding the 10 warning signs proposed by the Jeffrey Modell Foundation for the suspicion of PID, the most frequent were need for intravenous antibiotics to clear infections (19/26 = 73.08%), two or more pneumonias within 1 year (14/26 = 53.85%), and failure of an infant to gain weight or grow normally (12/26 = 46.15%). See Table 4 for the frequency of the 10 warning signs proposed by the Jeffrey Modell Foundation in the studied patients.

## 4. Discussion

Patients with PID in the present study had similar, as well as different, characteristics, compared to those reported previously in other countries. We found that the proportion of male and female is similar to that reported in national surveys in the USA, Australia, and Europe, with PID being more frequent in males [3, 7, 9]. The diagnostic delay reported in the population of the state of Guanajuato was shorter than that reported in a recent study in Mexico City [10] for patients with CVID (4.65 versus 12.5 years) and almost the same as that observed in a study of Iranian patients with CVID (4.65 versus 4.4 years) [11]. However, in a survey conducted in Republic of Korea, the time elapsed between the onset of clinical symptoms and PID diagnosis was only of 19 months [12]. We should try to decrease the time of delay in diagnosis, since this could diminish the number of complications attributed to PID. In fact, data show that the proportions of Iranian patients with CVID [11] and those in our study with bronchiectasis and chronic lung disease were of 30.4% and 30.76%, respectively. In contrast, such complications were considerably less frequent in the Korean population with PID (11.5%) [12], perhaps due to the lower delay in diagnosis as commented before. Mortality rate in the present study was similar to that reported in the national registry of PID in Korea (11.54 versus 9.8%) [12]. Looking at the bright side, only one patient (3.85%) was diagnosed after 16 years of age in the present survey, whereas the European internet-based patient and research database for PID shows that more than 21% of all registered patients were diagnosed at 16 years of age or later [3].

The spectrum of PID in the state of Guanajuato is similar to that observed in other studies. Antibody deficiency was seen in 65% of the patients, which is consistent with international reports of several research groups. For example, the proportion of patients with antibody deficiency in Europe is 54.82%, in Australia is 77%, in Korea is 53.3%, in Japan is 52.9%, and in Switzerland is 66.2% [3, 9, 12–14]. The second and third places in our study were occupied by the well-defined immunodeficiency syndromes and the congenital defects of phagocyte number and/or function, respectively; these findings are consistent with data reported in the European and Australian surveys [3, 9].

In the present study, all patients with CVID and XLA were under IRT. This proportion is higher than that reported in other studies. The 2007-USA national survey revealed that the proportions of patients under IRT with XLA were 67% and 13% in patients with CVID [7]. In Australia and New Zealand, the percentages of patients under IRT with XLA and CVID are of 88.9% and 58.6%, respectively [9]. The highest rates of IRT were reported in the 2006–2008 European database for PID, with a coverage of 92% for XLA and 85% for CVID [3]. The trough serum level, which is measured immediately before IgG is administered, should be at least 500 mg/dL and ideally within the normal range of healthy individuals (700–1600 mg/dL) [3, 15]. In fact, one study indicates that the rate of infection can be further reduced with a trough level of 900 mg/dL [16, 17]. The means IgG trough level in the ESID database were 685 mg/dL for CVID patients and 700 mg/dL for XLA patients [3]. In our study, the IgG trough levels were 1434.13 mg/dL for CVID and 1115.5 mg/dL for XLA patients. As all patients with XLA and CVID were under IRT and the trough levels of IgG were above the recommended by international guidelines, we can assume that the different institutions and their physicians are doing a good effort to improve the quality of life among their patients.

The overall consanguinity rate (3.85%) in the present study was very low, compared with that reported in other studies. In one study of Iranian patients with CIVD, a family history of immunodeficiency was recorded and first-degree parental consanguinity was observed in 72.4% [11]. The low consanguinity rate among patients with PID, reported in our study, might suggest specific genetic characteristics of patients with PID in the Mexican population. This fact is supported by the finding that no family history of PID among relatives of our patients was reported. In contrast, 23% of the patients in the national registry of PID in Republic of Korea had one or more family members with proven or suspected immunodeficiency [12].

In the present study we found that, until diagnosis, patients had a mean of 12.5 visits to the emergency room, 16.1 doctor's visits/year per patient, and 6.35 hospitalizations related to PID. Unfortunately, a late diagnosis may increase the number of visits to the emergency room, doctor's visits, hospitalizations, severe infections, and permanent sequelae in patients with PID. This situation has been described in other studies; for example, in the first national survey in the USA, most patients experienced two or more hospitalizations before diagnosis [8]. As we can see, in addition to the deleterious effect on health, there is considerable economic and psychosocial morbidity associated with these disorders. Fortunately, it has been shown that effective treatment can reduce significantly the burden of disease [8].

There is still much to be done in Mexico and our state. We need to know the real prevalence of PID in Mexico, and increase the level of suspicion among physicians to diminish the time of diagnosis delay, in order to improve the quality of life among patients with PID and their relatives.

## Figures and Tables

**Table 1:** General characteristics of patients with primary immunodeficiency diseases at reference and high-specialty hospitals in the state of Guanajuato.

	Study group *n* = 26
Gender (male/female)	15/11
Mean age at the onset of symptoms, years	3.46 ± 6.32
Mean age at the time of diagnosis, years	8.2 ± 10.93
Mean time in diagnostic delay, years	4.65 ± 6.95
Patients under 16 years at the moment of diagnosis, *n* (%)	25 (96.5%)
Overall mortality rate since diagnosis until the time of the study, *n* (%)	3 (11.54%)
Patients with complications secondary to PID, *n* (%)	10 (43.48%)
Mean number of hospitalizations per patient until diagnosis	6.35 ± 7.51
Mean number of visits to emergency room per patient until diagnosis	12.5 ± 15.2
Mean number of doctor's visits/year per patient	16.13 ± 7.82

**Table 2:** Spectrum of PID at reference and high-specialty hospitals in the state of Guanajuato.

PID	Study group *n* = 26	Method of diagnosis
Combined immunodeficiencies, *n* (%)	1 (3.85%)	Decreased numbers of lymphocytes and immunoglobulins levels associated with opportunistic infections
Complement deficiencies, *n* (%)	1 (3.85%)	Quantitative C1 inhibitor deficiency
Defects in innate immunity *n* (%)	1 (3.85%)	
Chronic mucocutaneous candidiasis	1/1	Phenotypic diagnosis: persistent mucocutaneous candidiasis
Congenital defects of phagocyte number and/or function	2 (7.69%)	
Chronic granulomatous disease	1/2	Dihydrorhodamine (DHR) flow cytometry test
Cyclic neutropenia	1/2	Low neutrophils count
Well-defined immunodeficiency syndromes, *n* (%)	3 (11.55%)	
Ataxia-telangiectasia	1/3	Syndromic features
Chromosome 22q11.2 deletion syndrome	1/3	FISH test for 22q11 deletion
Hyper-IgE syndrome	1/3	Syndromic features, NIH clinical feature scoring system
Predominantly antibody deficiency disease, *n* (%)	17 (65.38%)	
CVID	8/17	Low IgG and IgA and/or IgM
X-linked agammaglobulinemia	3/17	Mutation in BTK. Severe reduction in all serum immunoglobulin isotypes with profoundly decreased or absent B cells
Selective IgA deficiency	2/17	IgA decreased/absent
Isolated IgG subclass deficiency	2/17	Reduction in one or more IgG subclass
THI with normal numbers of B cells	2/17	IgG and IgA decreased

PID: primary immunodeficiency diseases; CVID: common variable immunodeficiency disorders; THI: transient hypogammaglobulinemia of infancy.

**Table 3:** Clinical data on etiological agents of infectious diseases in the studied group of patients.

No.	Gender	Diagnosis	Infectious diseases (number of episodes)	Etiologic agents	Comorbidity
1	Female	Combined immunodeficiency	Pneumonia (3), urinary tract infection (1)	*Burkholderia cepacia, E. Coli * *Candida albicans. *	Ichthyosis
2	Female	CVID	Pneumonia (4)	Nonisolated pathogens	Down syndrome, Hypothyroidism
3	Male	Selective IgA deficiency	Recurrent URTI	Nonisolated pathogens	None
4	Female	Ataxia-telangiectasia	Tonsillitis (3), gastronitestinal infection (1)	*S. pyogenes *	None
5	Male	CVID	Pneumonia (3), sinusitis (4)	*S. pneumonia *	None
6	Male	Selective IgA deficiency	Recurrent URTI	Nonisolated pathogens	Food allergy
7	Male	THI	Recurrent URTI	Nonisolated pathogens	None
8	Male	Isolated IgG subclass deficiency	Pneumonia (1), otitis media (5), sinusitis (1). Gastrointestinal infection (2)	*S. pneumoniae *	Hypertrophic cardiomyopathy
9	Male	Isolated IgG subclass deficiency	Otitis media (1), Sinusitis (3)	Nonisolated pathogens	None
10	Male	X-linked agammaglobulinemia	Pneumonia (5), sinusitis (3), otitis (5). Meningitis, recurrent URTI	*S. epidermidis,* *S. pneumoniae *	None
11	Male	X-linked agammaglobulinemia	Pneumonia (3), sinusitis (2)	*Klebsiella pneumoniae*, *Candida *sp., *S. pneumoniae *	Rheumatoid arthritis
12	Male	X-linked agammaglobulinemia	Pneumonia (2), sinusitis (3), pyoderma gangrenosum	*Pseudomonas* sp	Allergic rhinitis
13	Female	CVID	Pneumonia (8), sinusitis (2)	Nonisolated pathogens	None
14	Female	CVID	Gastrointestinal infection (4), pneumonia (3)	*Giardia lamblia *	Autoimmune thyroiditis, Hypothyroidism
15	Female	CVID	Gastro intestinal infection (5), pneumonia (2), intestinal tuberculosis	*Mycobacterium tuberculosis *	Arthritis
16	Male	CVID	Pneumonia (7)	Nonisolated pathogens	Allergic rhinitis
17	Female	CVID	Pneumonia (2), sinusitis (2)	Not available data	None
18	Female	THI	Recurrent URTI	Nonisolated pathogens	Down syndrome, IAC
19	Male	Chronic granulomatous disease	Pneumonia (10), skin abscesses, lung abscess	*Serratia marcescens*, *S. aureus*., *Stenotrophomonas maltophilia, E. Coli *	None
20	Male	Chronic mucocutaneous candidiasis	Persistent thrush, onychomycosis, recurrent URTI skin abscesses, varicella	*Candida* sp., *Klebsiella pneumoniae*, *Herpes zoster *	None
21	Female	Hereditary angioedema	URTI (2/year)	Nonisolated pathogens	None
22	Male	Hyper-IgE syndrome	Skin abscesses, pneumonia (1), recurrent URTI otitis (9)	*S. aureus *	Cow's milk allergy, GERD
23	Female	Chronic mucocutaneous candidiasis	Persistent thrush, onychomycosis, pneumonia (11)	*Candida* sp.	Hypothyroidism, cow's milk allergy, GERD
24	Male	Cyclic neutropenia	Periodontitis, recurrent URTI, pneumonia (3). Otitis (3), Sinusitis (1), gastrointestinal infection (2)	Nonisolated pathogens	None
25	Male	Chromosome 22q11.2 deletion	Pneumonia (4)	*S. pneumonia *	Cardiopathy, Pulmonary hypertension
26	Female	CVID	Pneumonia (4), UTI (2)	*Moraxella catarrhalis, S. viridans *	Allergic rhinitis

CVID: common variable immunodeficiency disorders; GERD: gastroesophageal reflux disease; IAC: interauricular communication; THI: transient hypogammaglobulinemia of infancy; URTI: upper respiratory tract infections; UTI: urinary tract infection.

**Table 4:** Frequency of warning signs proposed by the Jeffrey Modell Foundation in the studied group of patients.

Warning signs	Study group *n* = 26
Need for intravenous antibiotics to clear infections	19 (73.08%)
Two or more pneumonias within 1 year	14 (53.85%)
Failure of an infant to gain weight or grow normally	12 (46.15%)
Two or more deep-seated infections including septicemia	11 (42.31%)
Two or more serious sinus infections within 1 year	7 (26.92%)
Four or more new ear infections within 1 year	3 (11.54%)
Recurrent deep skin or organ abscesses	3 (11.54%)
Persistent thrush in mouth or fungal infection on skin	2 (7.69%)
Two or more months on antibiotics with little effect	2 (7.69%)
Family history of primary immunodeficiency	0 (0%)
